# Adherence to a guided walking program with amelioration of cognitive functions in subjects with schizophrenia even during COVID-19 pandemic

**DOI:** 10.1186/s13102-022-00440-2

**Published:** 2022-03-25

**Authors:** S. Mandini, M. Morelli, M. Belvederi Murri, L. Grassi, S. Masotti, L. Simani, V. Zerbini, A. Raisi, T. Piva, G. Grazzi, G. Mazzoni

**Affiliations:** 1 Department of Neuroscience and Rehabilitation, Center for Exercise Science and Sports, University of Ferrara, via Gramicia 35, 44121 Ferrara, Italy; 2 Department of Neuroscience and Rehabilitation, Institute of Psychiatry, University of Ferrara, Ferrara, Italy; 3 Public Mental Health Department, AUSL Ferrara, Ferrara, Italy; 4 Public Health Department, AUSL Ferrara, Ferrara, Italy; 5 Healthy Living for Pandemic Event Protection (HL-PIVOT) Network, Chicago, IL USA

**Keywords:** Schizophrenia, Physical activity, Mental disorder, Cardiovascular risk factors

## Abstract

**Background:**

Aim of the study was to enrol a group of individuals with schizophrenia in a long-term moderate-intensity physical activity program and to evaluate its effects on their cognitive functions and cardiovascular risk factors. An additional aim of the study was the comparison of the adherence to the physical activity program before and during the COVID-19 pandemic.

**Methods:**

Forty sedentary patients diagnosed with schizophrenia (mean age 46.4 ± 9.6) followed by the Public Mental Health Department of Ferrara were included in the study. 28 of them followed a 1-year walking program consisting of two guided walking sessions/week, while 12 maintained their sedentary lifestyle and followed the usual Cognitive Rehabilitation program. To the participants following the walking program VO_2_ peak and walking speed were assessed at baseline and at the end of the program. All participants were evaluated on blood pressure and anthropometric variable. Cognitive functions were assessed with the Screen for Cognitive Impairment in Psychiatry (SCIP) and with the Frontal Assessment Battery (FAB) questionnaires.

**Results:**

The 20 participants completing the walking program displayed significant improvements in cognitive functions (d_ppc2_ 0.35 for SCIP and 0.26 for FAB), with a positive correlation between SCIP score and the number of sessions attended (R = 0.86, *p* < 0.001), evident in the patients attending to at least 75 of the 100 walking sessions. Walking speed and VO_2_peak increased significantly and a decrease of body weight, BMI, systolic and diastolic blood pressure was also observed. The adherence to the walking program registered during Covid-19 period did not differ from that observed before the pandemic. The 12 CG (Control Group) patients maintaining the sedentary lifestyle did not display improvements of cognitive functions.

**Conclusions:**

The main finding of this study is the improvement of cognitive functions which is significantly related to the number of walking sessions attended by participants with schizophrenia. The walking program, guided by exercise specialists, proved to be an enjoyable activity for people with mental disorder feasible even during the COVID-19 pandemic.

*Trial registration* Retrospectively registered on ISRCTN as non-randomized trial (n. ISRCTN14763786).

## Background

Schizophrenia is a common and expensive public health problem. Individuals affected by schizophrenia experience severe and chronic levels of disability that derive from acute psychotic symptoms, as well as from cognitive impairments [1]. Moreover, their life expectancy is up to 15 years shorter than that of the general population [2]. Several studies have found that individuals with schizophrenia display a high prevalence of modifiable cardiovascular risk factors such as obesity, dyslipidaemia, smoking, hypertension, hyperglycaemia, physical inactivity and moreover, they have lower cardiorespiratory fitness than the general population [3–7]. Interestingly, cognitive impairment is often associated with the metabolic syndrome in schizophrenia [8].

Recently, adults with a schizophrenia disorder were associated to an increased risk also for the new Coronavirus disease (COVID-19) mortality [9]. If the stress related to the COVID-19 pandemic and corresponding public health measures worsens mental health in the general population, even more so it is possible that their impact might be even higher in people living with schizophrenia. In addition, COVID-19 infection itself may exacerbate symptoms in people with schizophrenia, as coronaviruses may be associated with psychotic symptoms through an immune-related mechanism [10]. The COVID-19 lockdown could engender disruption to lifestyle behaviours, thus impairing mental wellbeing also in the general population [11, 12].

According to the World Health Organisation, at least 60% of the general population does not follow the minimum guideline for health-related physical activity [13]. It becomes clear that individuals with schizophrenia, who are more limited in their ability to be physically active than the general population, will also demonstrate lower levels of physical fitness and consequently more susceptible for disease.

The World Health Organization (WHO) and the European Mental Health Action Plan 2013–2020 [14, 15] acknowledge the role of physical activity (PA) in mental health and encourage the inclusion of lifestyle modifications in education and treatment programmes for people with mental health conditions, delivered in primary health care settings. Several studies have identified physical activity as a powerful lifestyle factor that plays a critical role in preserving and even improving cognitive function through the lifespan [16].

People with mental illnesses tend to engage in less physical activity programmes than the rest of the population and experience a range of barriers to the engagement in PA such as pain, side effects of medications and negative symptoms [12–15].

As indicated by recent meta-analyses, while no difference in PA is detected relying on self-reported measures [14, 15, 17–21], studies based on accelerometry show a consistent, large reduction of PA in schizophrenia compared to the general population [22–25].

A meta-analysis of ten studies recently suggests that aerobic exercise might improve cognitive functions in people with schizophrenia [26]. Aerobic exercises (as cycling, treadmill and elliptical training) or anaerobic exercises like mild weight training and flexi bars were successful in improving the cognitive domain of working memory, social cognition and attention/vigilance [27]. However, the specific training characteristics contributing to cognitive improvements were not clarify [28].

If such findings were confirmed, they would be clinically relevant especially considering that few interventions are available to address the cognitive impairments associated with schizophrenia [25, 26]. Moreover, physical exercise, especially if maintained in the longer term, may be able to significantly reduce cardiovascular mortality while exerting positive effects on patient cognition. However, no study, to our knowledge has investigated the effects of long-term physical exercise on cognition in schizophrenia. We previously showed that a 6–12 months program of guided walking was followed by significant improvements of cardiovascular risk in a large population of sedentary adults [29–32]. The primary outcome of the present pilot study were: (i) to evaluate the feasibility and adherence of a long-term, moderate-intensity physical activity program for individuals with schizophrenia (ii) to evaluate its effectiveness on cognitive functions, (iii) to document the possibility of extending to these patients the reduction of cardiovascular risk factors that follow prolonged periods of physical activity and (iv) to compare between the adherences to the guided walking program before the COVID-19 pandemic and during the restriction imposed to contain the pandemic.

## Methods

### Study design

This was a 1-year pilot study of guided walking program compared to a usual cognitive rehabilitation program.

Sixty-seven sedentary participants with schizophrenia (aged from 25 to 70) referring to the Public Mental Health Department of Ferrara were recruited from December 2017 to December 2018. Follow up evaluations were completed in December 2019 but the walking program continues.

This study has been retrospectively registered on ISRCTN as non-randomized trial (n. ISRCTN14763786).

#### Inclusion criteria

In order to be recruited, patients had to be diagnosed with schizophrenia since at least one-year and on antipsychotic medications and with the same therapeutic regimen for at least three months before enrolment. In addition, they had to be free of symptomatic peripheral arterial occlusive disease and cardiovascular, pulmonary, neurological, metabolic, and orthopaedic disorders that could interfere with the walking activity.

**Fig. 1.**
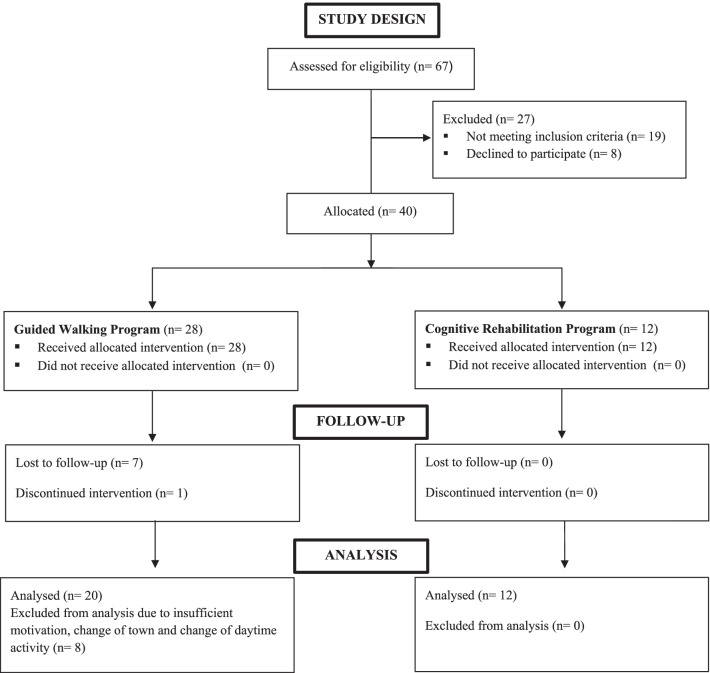
Study design, according to TREND statement

The eligible participants provided written informed consent and were subdivided to the Guided Walking Program (WG) or to the Cognitive Rehabilitation Program (CG) with an allocation of 2:1 (Fig. 1) basing on their willingness according to their presence in the Daily Centre in the walking day.

The investigators involved in data collection were not aware of the allocation list until the assignment. This study was approved by the Ethics Committee (number 22-13).

### Evaluations in both groups

#### Cognitive and executive functions

The Italian version of the Screen for Cognitive Impairment in Psychiatry (SCIP) [33] and the Frontal Assessment Battery (FAB) [34] were used for the assessment of cognitive functions.

The SCIP is a brief screening tool designed for evaluating immediate and delayed verbal learning, working memory, verbal fluency and processing speed.

The FAB consists of six subtests exploring conceptualization, mental flexibility, motor programming and sensitivity to interference, inhibitory control and environmental autonomy.

These tests proved to be reliable in identifying the cognitive impairments associated with schizophrenia.

#### Blood pressure

Arterial blood pressure was determined in the seated position after three minutes of rest, using a validated automatic sphygmomanometer (Omron M3) and averaging the values of three successive determinations taken with two-minute intervals. Participants were instructed not to carry out any physical activity in the 12 h before this evaluation. Participants presenting with a systolic blood pressure above 130 mmHg were enrolled following the authorization of the family doctors.

#### Anthropometric measurements

Height and weight were measured, and BMI was calculated. Waist circumference was measured using standard procedures [35].

Values for both groups are reported in Table 1.

**Table 1:** Characteristics of the study population at enrollment

	Walking group (n = 28)	Control group (n = 12)	Comparison between groups
Age (yrs)	44.3 ± 8.7	48.8 ± 12.6	*P* = 0.14
Gender (Male/Female)	15/13	8/4	*P* = 0.84
SCIP score	64.9 ± 11.3	68.1 ± 13.2	*P* = 0.38
FAB score	14.8 ± 1.2	15.1 ± 2.6	*P* = 0.89
Weight (kg)	87.6 ± 16.1	85.6 ± 13.1	*P* = 0.71
BMI (kg/m^2^)	30.8 ± 5.3	30.2 ± 3.6	*P* = 0.72
Waist circumference (cm)	107.7 ± 13.1	105.4 ± 7.7	*P* = 0.59
Systolic blood pressure (mmHg)	125.6 ± 11.8	128.8 ± 12.8	*P* = 0.49
Diastolic blood pressure (mmHg)	83.0 ± 5.4	82.9 ± 5.8	*P* = 0.96
Walking speed (km/h)	4.8 ± 0.5		
Estimated VO_2peak_ (ml/kg/min)	28.3 ± 6.9		

### Evaluations in the guided walking group

#### VO_2_peak determination

VO_2_peak was indirectly determined using the 1-km walking test (1 k-WT) previously described [36]. Five minutes of slow walking preceded the test. Participants were instructed to select a pace that they could maintain for 10 to 20 min at a moderate perceived exercise intensity, (11–13 on the 6–20 Borg scale) [37]. Heart rate was monitored continuously using a Polar Accurex Plus heart rate monitor (Polar Electro, Kempele, Finland). The equation for VO2 peak determination considering age, BMI, HR and time to complete the 1 k-TWT was then applied [36].

All measurements were carried out at baseline and after 1-year, at the end of the walking program.

### The guided walking program

Two walking sessions were organized each week and guided by exercise physiologists. The walking sessions were carried out outdoors on flat ground. Their duration varied from 45 to 60 min and the distance covered ranged from 4 to 6 km.

Some increments of walking speed and duration occurred within the first months: they were not forced but chosen spontaneously by the participants.

To motivate participants and enhance compliance, monthly meeting on the importance of regular physical activity were held.

### The cognitive rehabilitation program

Participants allocated in the control group were enrolled in a 1-year program of usual cognitive rehabilitation followed by the Psychiatric Rehabilitation Centre of the Healthcare District of Ferrara, Italy. Specialized psychiatric therapists conducted this program with groups of 5–10 participants, based on a Cognitive Remediation model. Each session consisted in welcoming participants, reviewing tasks that were conducted in the last meeting, and assignment of new pen-and-pencil cognitive and metacognitive tasks. Tasks included games and exercises (e.g. sodoku, crosswords), as well as discussions aimed at improving short- and long-term memory (e.g. repeating sequences of words and numbers), executive functions, social cognition, with progressive adaptation of task difficulty. The total number of sessions was 50 for a duration of 12 months.

### Statistical analysis

The assessed variables (systolic blood pressure, diastolic blood pressure, weight, BMI, waist circumference, VO_2_peak, walking speed and SCIP score) and their relative changes are expressed as mean ± standard deviation. The normal distribution was assessed with the Kolmogorov–Smirnov test. Differences between baseline and study-end values were analyzed using paired samples Student’s *t*-tests. The Wilcoxon test was used to assess differences in baseline values of the SCIP score between the WG and the CG. Further, we explored the relationship between adherence (number of attended sessions) and cognitive changes using Pearson correlation, followed by non-linear regression analysis, including a quadratic term. A stepwise multivariate regression analysis was used to assess whether age, gender, number of walking sessions attended and change in weight, BMI, walking speed and VO_2_peak were related to changes of SCIP total scores after 1-year of guided walking.

As measures of the effect size, we report the values of the Standardized Mean Difference (Hedges’ *g*) for pre-post differences in each group test scores, as well as the d_ppc2_ sensu Morris [38, 39], that is, using the pooled pretest standard deviation for weighting the differences of the pre-post-means.

Given the exploratory nature of the study, the level of statistical significance was set at *P* < 0.05 for all tests. Statistical analyses were performed using MedCalc for Windows, version 15.0 (MedCalc Software, Ostend, Belgium).

## Results

Twenty-seven participants were excluded for ineligibility and eight declined to participate.

Twenty-eight participants with schizophrenia were included in the guided walking group while twelve followed the usual Cognitive Rehabilitation program.

### Baseline values

The baseline characteristics of the participants are reported in Table 1.

No differences in age, gender, anthropometric characteristics, arterial blood pressure values and cognitive function level were found at baseline within the groups.

#### Walking group

Of the cardiovascular risk factors considered, smoking (63.4%) and being overweight with a BMI above 25 (87.8%) were extremely common. One participant had type 2 diabetes, while seven had hypertension with a systolic blood pressure above 130 mmHg. The baseline SCIP total score was 64.9 ± 11.3 while the FAB score was 14.8 ± 1.2. Estimated VO_2_peak was 28.3 ± 6.9 ml/kg/min: the average walking speed was 4.8 ± 0.5 km/h.

#### Control group

Also in this group, smoking (75%) was extremely common. The SCIP total score was 68.1 ± 13.2 and the FAB score was 15.1 ± 2.6.

### Values at 1-year

#### Walking group

Eight participants left the walking program within the first six months after enrolment, for insufficient motivation (n = 4), change of town (n = 1) and change of daytime activity (n = 3) and were excluded from the analyses. Age, anthropometric and functional variables of the dropouts were not significantly different from those of the patients completing the study.

Twenty participants (7 females and 13 males) completed the project. Their drug therapy did not change during this period. No participant suffered injuries or experienced side effects during the program.

The 1-year program encompassed 100 walking sessions (averaging two sessions per week). Session duration and distance were increased progressively up to 60–70 min and to five kilometres during the first four months, while they were kept constant thereafter.

Participation in the walking program of completers ranged from 21 to 93 sessions (mean of 67.8 SD: 20.6) of the 100 that were organized.

**Fig. 2.**
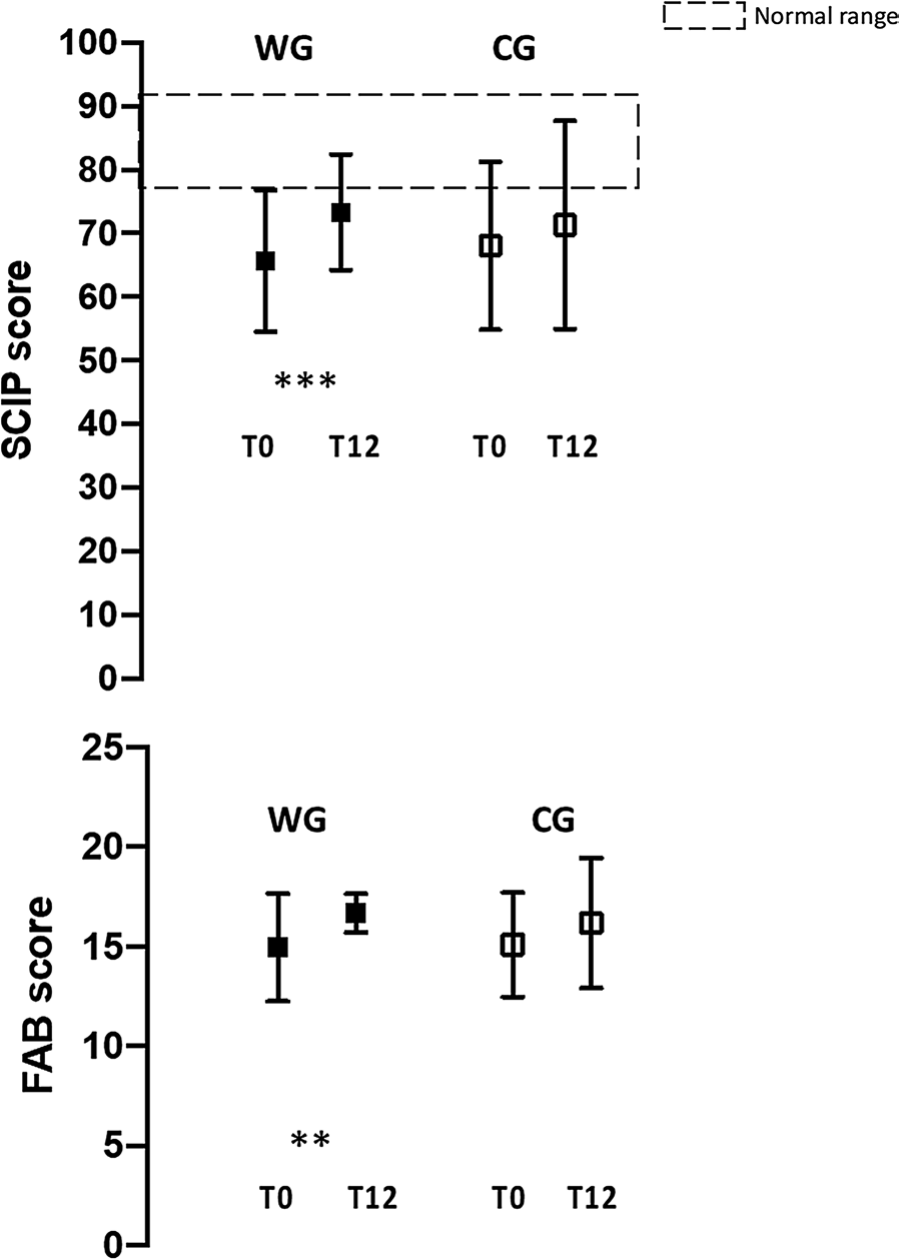
SCIP and FAB score variations in the Guided Walking group (WG) and in the Control Group (CG)

A significant improvement of the total score of SCIP (*P* < 0.001) and FAB (*P* < 0.001) was evident (Fig. 2).

**Fig. 3.**
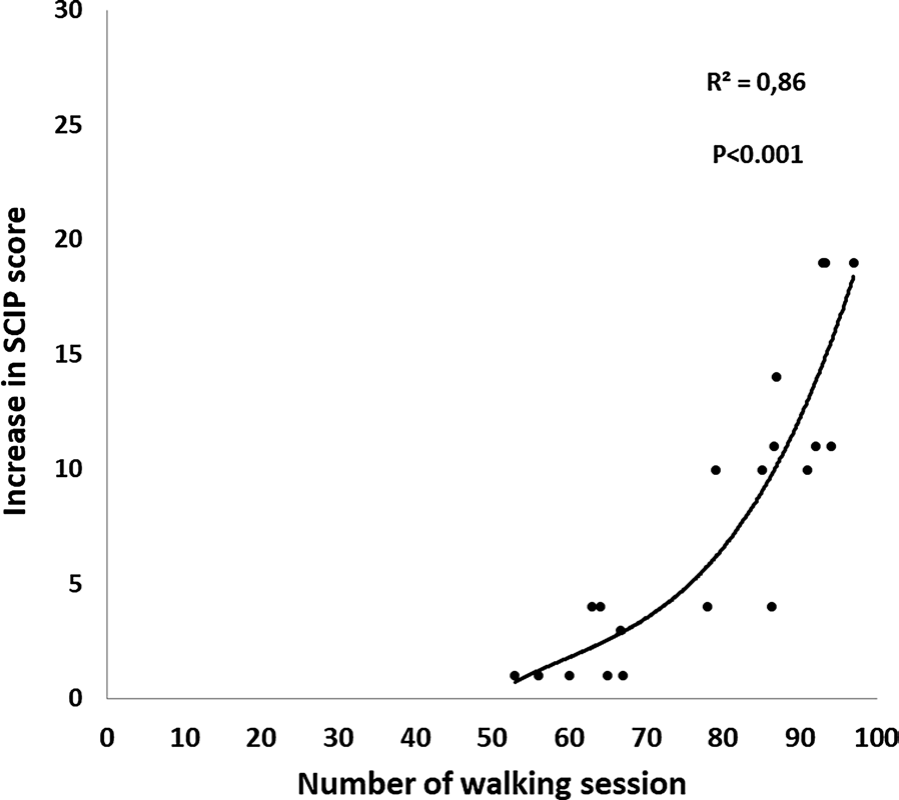
Relationship between number of walking session attended and the increase in SCIP score after 1-year of guided walking

Multiple regression analyses revealed that the increase in SCIP total score was associated with the number of walking sessions attended (*P* < 0.001) and not with age, gender and nor to changes in body weight, BMI, VO_2_peak and walking speed. The relationship between the number of walking sessions attended and the improvement of the SCIP score is presented in Fig. 3. The relationship is described by a linear regression with an R^2^ = 0.86 (*P* < 0.001) adjusted for age and gender.

The results in cognitive functions obtained after the 1-year walking program are shown in Table 2.

**Table 2:** Change in cognitive function after 1 year among walking group and control group participants

	Walking group (n = 20)			Control group (n = 12)		
	Baseline	1-year	*P*	Baseline	1-year	*P*
SCIP score	64.9 ± 11.3	73.2 ± 9.4	< 0.001	68.1 ± 13.2	71.3 ± 13.4	0.50
FAB score	14.8 ± 1.2	16.7 ± 1.0	< 0.001	15.1 ± 2.6	16.2 ± 3.3	0.18

*FAB* frontal assessment battery, *SCIP* screen for cognitive impairment in psychiatry

A significant reduction of body weight, BMI, systolic and diastolic blood pressure was observed, as well as increases of VO_2_peak and walking speed (Table 3).

**Table 3:** Values of the variables considered at baseline and after 1-year of guided walking. Values are reported as mean ± standard deviation

	Walking group (n = 20)			Control group (n = 12)		
	At baseline	After 1-year	*P*	At baseline	After 1-year	*P*
Age (yrs)	44.0 ± 8.7	–		48.8 ± 12.6	–	
Gender (Male/Female)	15/13	–		8/4	–	
Weight (kg)	87.6 ± 16.1	84.9 ± 14.5	0.03	85.6 ± 13.1	85.9 ± 12.9	0.43
BMI (kg/m^2^)	30.8 ± 5.3	29.8 ± 4.5	0.04	30.2 ± 3.6	29.4 ± 3.6	0.23
Waist circumference (cm)	107.7 ± 13.1	105.8 ± 10.7	0.70	105.4 ± 7.7	106.2 ± 8.0	0.06
Systolic blood pressure (mmHg)	125.6 ± 11.8	117.7 ± 7.7	0.001	129.0 ± 12.8	128.8 ± 11.6	0.80
Diastolic blood pressure (mmHg)	83.0 ± 5.4	78.7 ± 6.4	0.02	82.9 ± 5.8	81.7 ± 6.2	0.33
Walking speed (km/h)	4.8 ± 0.5	5.1 ± 0.7	0.01			
Estimated VO_2_ peak (ml/kg/min)	28.3 ± 6.9	32.2 ± 4.9	0.01			

#### Control group

The cognitive rehabilitation program was well tolerated and completed by all the twelve participants enrolled. No participant suffered injuries or experienced significant side effects during the program. Their drug therapy did not change during this period. The rate of participation of the CG ranged from 20 to 41 sessions of the 50 (mean of 53.4 SD: 7.0) that were organized. After 1-year the total score of SCIP and FAB did not change significantly (Fig. 2).

Overall, the WG was associated with a relative greater improvement than the CG in both SCIP and FAB scores; this corresponded to effect sizes (d_ppc2_) of 0.35 for SCIP and 0.26 for FAB (Table 2).

#### Physical activity maintenance during COVID-19 pandemic

Given the positive results on exercise capacity and cognitive function, the walking program continued even after the one-year study follow-up.

**Fig. 4.**
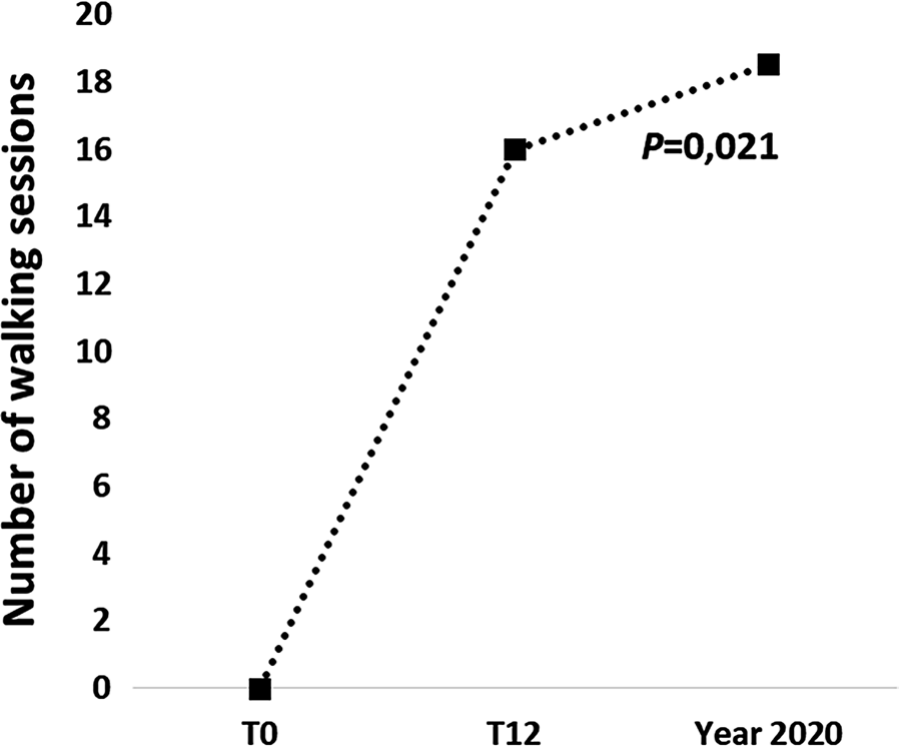
Number of walking session organized from baseline (T0), 1-year follow up (T12) and during the Covid-19 pandemic (year 2020)

In the 2020, the restrictions due to the COVID-19 pandemic have imposed the suspension of the walking program in the months of April and May 2020. Despite this interruption, there was no significant difference in terms of attendance at the walking sessions in year 2020 compared to 2019. However, due to the closure of gyms, twenty-one new participants chose to follow the walking program during the COVID-19 pandemic with a significant increase in the walking sessions organized (*P* = 0.02, Fig. 4).

## Discussion

### Adherence to the guided walking program

The walking activity proposed in this study, has been effective in involving these individuals in a 1-year physical activity program. The adherence rate was high (67.8%) especially when one considers the overall low levels of adherence of individuals with schizophrenia to several psychosocial programmes or drug treatments previously studied [19].

These considerations lead us to believe that their engagement to the project was favored by the presence of exercise physiologists supervising the activity and by the fact that walking session has been carried out in-group. Such characteristics have been previously associated with a greater adherence to guided exercise programs in depressed patients [40].

Moreover, the guided walking program proposed proved to be a feasible activity even during the restrictions due to the COVID-19 pandemic where all other physical activities were suspended, and the gyms closed. People who previously attended other sports activities, suspended due to the pandemic, chose to follow the walking groups in order to continue to be physically active.

### Adherence to the cognitive rehabilitation program

Although the cognitive program was less demanding in terms of weekly e engagement than the guided walking project, the adherence rate recorded by the individuals with schizophrenia allocated to this program was of 53%, in line with the percentages reported in the literature [19].

### Improvement of the cognitive functions

At baseline, the SCIP score of the subjects with schizophrenia enrolled in the study were well below Italian normal values (86.0 ± 7.0) reported by Belvederi Murri et al. [41, 42] with only 10 of the 40 participants enrolled presenting values within the normal range.

At baseline, the SCIP and FAB scores documented in the individuals enrolled in the WG e CG groups were superimposable. The scores, both for SCIP and FAB questionnaires, significantly increased only in the individuals following the walking program.

The relevant observation of this study is the significant correlation between walking sessions attended and improvement in cognitive functions (detected through the SCIP questionnaire): the improvement was documented only in the participants entering at least 1.5 sessions per week (Fig. 3). Similarly, Kimhy et al. have previously examined the relative influence of exercise duration, frequency, and intensity on cognitive improvements following a 12-week exercise program in schizophrenia and suggest that cognitive improvement is associated with the intensity of training [24]. Our observations are also in line with the results of previous studies indicating that the amount of whatever exercise performed by individuals with mental illnesses, including schizophrenia, is a significant predictor of cognitive improvements [24, 43]. Most of the interventions considered in the meta-analysis of Firth and co-workers [23] varied in physical activity type and duration (8–12 weeks long, with 2 or 3 sessions per week). Our results are also in line with those presented by Chekroud et al. [40] that have stressed not only the physical improvement but also the mental health benefits of regular physical exercise, especially when performed in-group and supervised by exercise physiologists.

### Improvement of cardiovascular functions

Cardiovascular diseases and diabetes represent the highest proportion of all natural causes of death in populations with severe mental illnesses. The 1-year guided walking program has been followed by an improvements in cardiovascular function as indicated by the increase of VO_2_peak assessed by the 1-km walking test [36]. This evaluation has proven to be an effective and well-tolerated submaximal test for VO_2_peak determination also in individuals with schizophrenia, considering also its feasibility on the 500 and 200 m. [44, 45] These results are in line with what previously demonstrated in sedentary and hypertensive adults [29–32]and in individuals affected by cardiovascular diseases in which the increase of exercise intensity is associated with a reduction in mortality and hospitalization [46, 47].

Walking activity, guided by exercise physiologists and performed in group can be considered an effective strategy to counteract the sedentary behaviour of individuals with schizophrenia, with a consequent improvement of their cardiovascular function and reduction of their mortality rate and, last but not least, an increment of the rate of their social inclusion.

## Conclusion

The main finding of this study is the improvement in cognitive functions documented in the participants completing the walking program with a significant correlation between the improvement and the number of walking sessions attended, evident only in the patients who attended to at least 1.5 sessions/week.

It can be hypothesized that walking programs guided by exercise physiologists and performed in-group may be effective in counteracting the sedentary behaviour of individuals with schizophrenia and may lead to the improvement of their cardiovascular function, to a reduction of their mortality rate and increment the rate of their social inclusion.

The maintenance of regular physical activity even during the COVID-19 pandemic may have helped the individuals with schizophrenia to preserve the cognitive and functional improvements obtained following the 1-year guided walking program.

Walking as (it) a simple and economic activity, does not requiring special equipment and can be practiced while maintaining the social distancing required by the current health emergency.

In order to confirm the results obtained including the anthropometric amelioration, a study with a larger population is in program.

### Limitations of the study

A study with a larger population is in program in order to verify the possibility to translate these preliminary positive results into a clinical significance. Future studies should also investigate the correlation between walking and every subdomain of cognitive questionnaire.

## Data Availability

The dataset used and analysed during the current study are available from the corresponding author on reasonable request.

## Abbreviations

CG: Control group
FAB: Frontal assessment battery
SCIP: Screen for cognitive impairment in psychiatry
VO_**2**_peak: Maximal oxygen consumption
WG: Walking group
